# The Distribution of Medical Officers in the Expeditionary Force

**Published:** 1915-10-02

**Authors:** 


### THE ROYAL ARMY MEDICAL CORPS.

### The Distribution of Medical Officers in the Expeditionary Force.

One of the many problems of administration in the Royal Army Medical Corps at work in the theatres of war is the proper distribution of medical officers so that as many as possible are allotted the kind of work which they are best fitted to perform. When the Expeditionary Force left England in August of last year it had been impossible during the haste of mobilisation to undertake any distribution except upon a purely chance basis: few of the specialists in the R.A.M.C.—as, for example, surgeons, sanitary experts, bacteriologists, and so forth—could be allotted to appointments connected with their specialty, and many men found themselves carrying out duties quite other than those in which they had specially trained themselves. Nor was the course of events during the first three months calculated to allow of any rearrangement in this respect. The thirteen days' retreat, the counter-advance across the Marne and its check upon the Aisne, the move to Flanders, and the desperate fighting there, were not exactly the times for nice adjustments in distribution: every man had to turn his hand to that which lay nearest and do it as best he could. All during that time, too, a steady stream of new blood was pouring into the R.A.M.C.; Special Reserve and the temporarily commissioned were being sent out in large numbers to replace casualties and fill up gaps. In very few cases was or could any attempt be made to classify the medical officers thus arriving, with a view to deciding the tasks for which their character, training, and experience best fitted them: and one way and another a certain number of square pegs inevitably got into round holes. That the interests of the Service so rarely suffered to any appreciable extent is due as much to the adaptability and practical nature of the English mind as to any special forethought on the part of those on whom the organisation of the Medical Service devolved. In later months a gradual sifting and sorting of the *personnel* has taken place, and the result is that a good many of the errors of distribution which at first made themselves evident have been corrected, with advantageous results to the military machine. It might be too much to say that the old haphazard method has been abandoned altogether, but from all accounts there is a marked improvement.

### The Two Ideals of Medical Officer.

Broadly speaking, the qualities most required of junior medical officers (those not employed on administrative work) of the R.A.M.C. are quite different at the Front and on the lines of communication respectively. At the Front the most necessary qualities are robust physical health, ability to "rough it" and to ride, courage under fire, and plain ordinary commonsense. Professional efficiency is naturally a good thing to have; but as the treatment is not as a rule much more than first-aid intelligently applied, experience of higher medicine and surgery is by no means essential. To sum it up, young, strong, willing, plucky fellows are what the field ambulances and the regimental establishments chiefly want.

On the lines of communication the requirements are not quite the same. It is immaterial, for instance, whether the medical officer is a horseman or not: it is less essential, though still desirable, that he should be of robust physique, because he will in all probability have a house or a tent to live in, and not much more exposure to the weather to undergo than in ordinary civil practice in England. It does not much matter what his age is; indeed, middle-aged men may be actually at a premium provided they have kept themselves up to date in every way. Professional efficiency, in fact, is very much more necessary than at the Front, where purely military qualities are what is chiefly required. On the lines of communication there is ample scope for the surgeon, the physician, the bacteriologist, the radiologist, the ophthalmologist, the neurologist, and other specialists; as well as for the man with all-round experience and plenty of it. Last of all there is another factor to be thought of—namely, the domestic condition of the medical officer: the Front is *par excellence* the place for the bachelor, the lines of communication for the married man. It is not for one moment suggested that married members of the medical profession in the R.A.M.C. are not so brave or have not done so well at the Front as the unmarried. On the contrary, a very large proportion of those who laid the foundation of the R.A.M.C. reputation for valour in this war during the first six weeks of the campaign were married men, many of them middle-aged: it is said that in a certain field-ambulance mess in those days ten members out of twelve were married men, and eight of the twelve were over forty years of age. But there is no denying that the young bachelor is more apt for the imminent deadly breach than the middle-aged married man, and it is legitimate to remember this distinction in distributing medical officers provided the interests of the Service are not in the slightest degree prejudiced thereby.

### Other Considerations.

The broad lines along which distribution should ideally be carried out have been indicated; but it must not be supposed that these are easy to translate into practice. Some quite young men are expert surgeons, and of more use on the lines of communication than older men whose professional equipment is of a less high standard. Other quite young men have less dash, less courage, less hardihood and toughness than some men old enough to be their fathers, and are in consequence less fitted for service at the Front. Some bachelors are more concerned for their personal safety than married men: it is not universally true that "Down to Gehenna or up to a throne, He travels the fastest that travels alone." And there are other disturbing influences such as the thirst for glory, which induces many men whose talents really best fit them for the lines of communication to volunteer for the Front. This is especially the case with young surgeons, who frequently get restive after a few weeks or months in base hospitals, and are discontented until they get themselves packed off to the trenches. It is seldom of any avail to point out to such keen young doctors that the work they do in hospital is of more value to the Army than that which they can do at the Front. They only half believe the statement, until actual experience convinces them that it is true; and they are often moved by a fear of what their friends at home will think of them if they do not go "under fire." As a matter of fact the vast majority of people in England seem to have no idea of the distinction between the Front and the lines of communication, and one often hears (in England) surgeons and others spoken of as "at the Front" who are no further from home than Boulogne or Havre.

### Casualty Clearing Stations.

It has been indicated that system now governs the distribution of medical officers, instead of the blind chance of a year ago; but occasionally grumbles still reach home; thus one hears of a London operator of many years' experience being at the Front as attached medical officer to an artillery brigade, a very pleasant billet indeed for anybody, but one which exacts the very minimum of professional skill for its performance. Here is an instance—if the report be true—of a good man absolutely wasted. Lately, by the way, a tendency—and a very proper one—has been felt towards undertaking more operations in the clearing stations than were formerly encouraged or permitted; indeed, it was understood at the beginning of this year that the reason for altering the old name of "clearing hospital" to "casualty clearing station" was to emphasise the temporary and transitional nature of the stay therein of the wounded and the official disfavour of conducting major operations in them. This seems now to be changed, and if the clearing stations are to approach nearer to hospitals in their functions, it is obvious that they will require rather more expert surgeons than were formerly allotted to them. We hear from various sources of instances in which this is being done; a study of the *Lancet* and the *British Medical Journal* is sufficient to show that quite a lot of ambitious surgery is being done in the clearing stations.

There is one element in the supply and distribution of medical officers which works strongly in the right direction, and that is the system of reinforcing. Groups of medical officers fresh from England arrive at frequent intervals at base ports in France, and are at once allotted by the local head of medical affairs to the units (general and stationary hospitals for the most part) in his command. When a call from the Front comes for a stated number of officers to fill gaps, the chiefs of the hospitals are called upon to supply the necessary men. They are fortunately allowed complete discretion as to whom they send, and so it happens that those whose services are found to be of value at the base are retained, and those who are thought to be more suitable for the Front are sent on. This results, after a few weeks or months, in a high level of efficiency among the staffs of the hospitals.

### Front *v.* Base.

One of the reasons why a considerable number of keen and able medical officers go to the Front from the base at their own request has already been suggested—namely, the idea that service in moderate comfort at the Front, especially "under fire," is more honourable than service at the base in ease and almost in luxury. The idea is natural enough, though based upon sentiment rather than reason, and it is perhaps encouraged by the customs and management of the Service. Thus leave to England is far more liberally allowed to those at the Front than to those at the base. The idea is, no doubt, that those who are bearing the burden of constant exposure to shell and rifle fire, to say nothing of mines, poison gas, flame projectors, etc., are more in need of a few days off than those in safety at the base. This argument is sound enough as applied to fighting troops, but is of far less weight in respect of the R.A.M.C. It perhaps applies to medical officers attached to infantry units, but certainly much less so to those of artillery brigades, ammunition columns, divisional trains, field ambulances, and cavalry field ambulances. In fact, it probably happens that medical officers, especially surgeons, in base hospitals live a far more strenuous and exacting life than do most of those who are technically "at the Front," and are far more in need for their health's sake of brief holidays. Another point of difference is in rewards and distinctions. It is most unusual for any medical officer (under the rank of lieut.-colonel) to be even mentioned in dispatches for work at the base, whereas decorations and mentions are issued in moderate profusion to those at the Front. It is, no doubt, inevitable and right that the Front should get the lion's share of these much-sought-for honours, because it is there that deeds of heroism in danger are alone performed. But the point is that many of the rewards distributed among those at the Front are not issued for deeds of gallantry, but merely for efficient service, and it is, therefore, not just that efficient service at the base should pass as unrecognised as it does. Nay, it would be nearer the truth to say that there is a great deal of unrecognised service at the base which is much more efficient and much more useful to the Army than that which at the Front is sometimes mentioned and decorated. So long as this disproportion of credit is allowed to exist, so long will there be a tendency for medical men whose services would be more usefully employed at the base to volunteer for the Front; and to grant such applications may tend to make the organisation of the medical service fall short of quite the highest ideal, closely though it does admittedly approach to it in most respects.

